# Ordered Porous Electrodes Obtained Using LIFT for Electrochemical Applications

**DOI:** 10.3390/ma16020596

**Published:** 2023-01-07

**Authors:** Korbinian Rager, Bo Tang, Christian Schneemann, Alexandra Dworzak, Mehtap Oezaslan, Andreas Dietzel

**Affiliations:** 1 Institute of Microtechnology, Technische Universität Braunschweig, 38124 Braunschweig, Germany; 2 Technical Electrocatalysis Laboratory, Institute of Technical Chemistry, Technische Universität Braunschweig, 38106 Braunschweig, Germany

**Keywords:** LIFT, 3D printing, porous ordered metal electrodes, electrochemically active surface area, roughness factor

## Abstract

Numerous synthetic techniques for the fabrication of porous metal electrodes were developed in recent decades. A very promising and facile route is the 3D printing of structures, which can be designed directly on the computer first. However, the current techniques allow structures to be printed with a resolution down to 20 µm, which is still quite rough regarding tuning the pore distribution and diameter of electrode materials for potential applications. For the first time, a laser-induced forward transfer (LIFT) process was used to 3D print metal voxels on a solid surface, resulting in a porous electrocatalytically active gold (Au) electrode film. Porous Au electrodes produced using LIFT showed an increase in the electrochemically active surface area (SA) by a factor of four compared with a sputtered dense Au film when characterized using cyclic voltammetry (CV) in Ar-saturated 0.1 M KOH. Therefore, the LIFT process can be considered very promising for the printing of ordered porous electrodes with high surface areas for electrochemical applications.

## 1. Introduction

The production of structured layers of metal or metal compounds is an essential element in the manufacture of microsystems and microsensors. These typically planar structures must be generated with high accuracy and resolution in a reliable production process. There are well-established micro-manufacturing techniques, such as the photolithographic structuring of thin metal layers. However, when it comes to rapid prototyping, maskless techniques, such as ink-jet or 3D printing, are advantageous but have not yet reached the resolution and reliability of photolithography [1]. An advantage of additive printing technologies is also that the sensitive base substrates are not exposed to aggressive chemicals, such as those used for selective removal. The processing becomes even more challenging when structured elements made of porous materials are to be introduced, which are required for multiple applications, such as separators, sensors, filters and electrodes [2]. For use in a lithium-ion battery cell, porous electrodes can help to accelerate charge and discharge times by reducing the ion diffusion time due to the smaller diffusion path in this porous matrix [3].

The state of the art includes different techniques for the production of porous metal electrodes that result in the stochastic arrangement of pores. An easy-to-adapt approach is the 3D printing of structures that were previously designed on the computer. A suitable variant of 3D printing is DMLM (direct metal laser melting) technology [4]. Different metals can be used, such as stainless steel or titanium. This technique allows structures to be printed with a resolution of down to 20 µm, which still is quite coarse for porous electrode fabrication. Another disadvantage is that during production, the whole device has to be immersed in the metal powder. Another approach to the production of porous electrodes that allows for much smaller pore sizes but not appearing as an ordered structure as in 3D printing is the production of nanofelts. These metal felts are produced using melt-spinning and sintered metal processes [5]. Wires in the nanometer range can be obtained and pressed together to ensure electric conductivity. The disadvantage of these nanofelts is that they can only be produced as bulk material, which must then be cut and contacted. Thus, a scalable micro-production process cannot be realized in this way.

Moreover, porous thin films of closely packed layers of particles made of SiO_2_, polymers or other materials, ranging in sizes from 60 to 1500 nm, were produced by evaporating the fluids of particle suspensions [6,7]. Similar thin films made of particles could be realized using the Langmuir–Blodgett method. These structures made of dielectric particles were finally coated with gold via electroplating, thereby forming porous gold electrodes. An even higher porosity could be achieved when particles were dissolved after gold electroplating [8,9]. As an alternative, thin layers of mixed metallic pastes (Au + Al) were sintered to remove polymer components and then etched with nitric acid to dissolve the silver to form porous gold electrodes [10]. All these methods involve many steps that include exposure to potentially aggressive chemicals.

**Figure 1:**
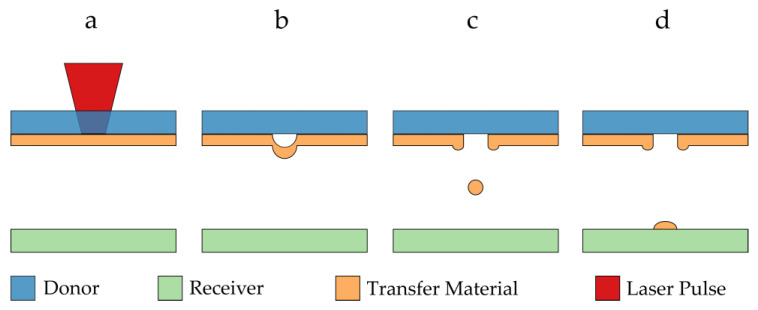
Schematic illustration of the basic principle of the LIFT process: (**a**) a laser pulse is focused on the interface between a transparent donor substrate and transfer material; (**b**) a gas pocket develops, expands and accelerates the transfer material away from the donor substrate; (**c**) a certain volume of material is ejected from the donor; (**d**) the material deposits on the receiver substrate to form a voxel at a predefined position.

An approach that has so far not been discussed in the context of porous electrode fabrication is the LIFT (laser-induced forward transfer) technique. As shown in Figure 1, the LIFT technique is a selective, maskless direct-printing process, where a transparent donor substrate carrying a thin film of material to be transferred is irradiated with single laser pulses. The absorption of laser light at the material interface leads to an internal ablation whereby part of the transfer material changes into a gaseous state (Figure 1a,b). The enclosed gas volume expands very quickly and locally transfers kinetic energy to the remaining non-gaseous transfer material, which is locally ejected toward a receiver substrate (Figure 1c,d). A large variety of materials can be used for this transfer, including both liquids and solids. Further, various LIFT process variants were reported in recent years, for which a good overview is given in [11].

Further developments in 3D printing based on the LIFT technique were used to form small metallic structures, such as coatings, constructional elements or even thermocouples [12,13]. Spheres down to 180 nm [14] were already LIFT-printed. Here, for the first time, the LIFT method was used to transfer well-defined metal volumes with each laser pulse, which built up a metal electrode with an ordered porous structure on the receiver substrate.

## 2. Materials and Methods

### 2.1. Laser Setup and LIFT Stage

For the LIFT experiments, a laser microstructuring system (MicroStruct C from 3D-Micromac, Chemnitz) equipped with a femtosecond laser source of Yb:KGW (Pharos Laser from Light Conversion, Vilnius, with a maximum average power of 15 W) and a minimum pulse duration of 224 fs was used. It provides emission at a basic wavelength of 1030 nm. A gold layer of 300 nm (as the donor metal layer) absorbs only about 3% of the incident light at 1030 nm. The repetition rate of the laser pulses could be set to either 100 kHz or 600 kHz, while the total output power remained constant. The pulse energy was adjusted using a high-voltage pulse picker. The laser beam was directed through a scan head with two galvo mirrors and focused using a telecentric F-theta objective on the thin film donor material. For beam shaping, a Ø 1.5 mm pinhole was placed in front of the focusing lens, which changed the beam profile from Gaussian to a top hat shape. Alternatively, a beam expander was applied, which doubled the beam diameter in front of the lens, leading to a smaller beam diameter in the focal plane.

**Figure 2:**
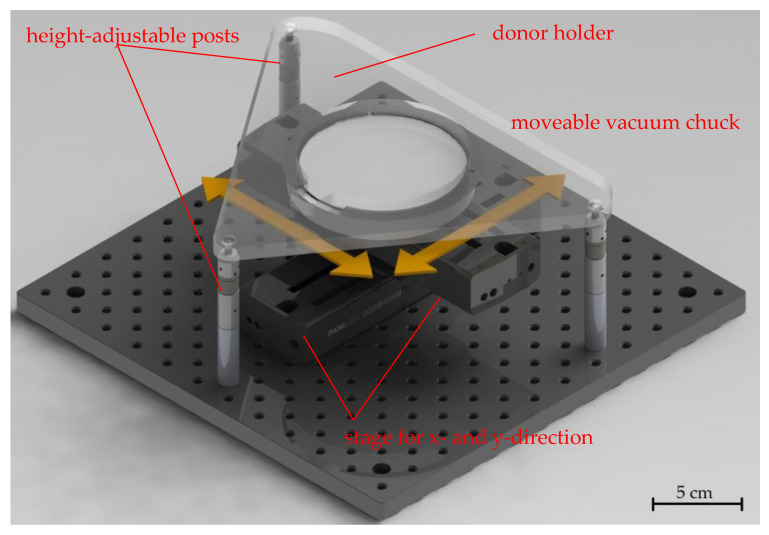
Annotated image of the custom-built LIFT stage with arrows indicating the axis directions for the motion of the vacuum chuck. The receiver was placed on the chuck, while the donor was held by the structure above. The distance between the donor and receiver could be adjusted using three height-adjustable posts. The parallelism between the substrates was secured and the distance could be kept constant during the LIFT process.

A customized LIFT stage that was introduced in previous work [15] can be seen in Figure 2. It was used to ensure an independent movement of the receiver substrate relative to the donor substrate. The stage consisted of two motorized axes (Thorlabs Inc. (Newton, NJ, USA), DDSM100) for the x- and y-directions connected with perpendicular orientations on which a porous ceramic holder was mounted, which held the receiver substrate via vacuum suction. A carrier plate for the donor substrate was mounted on three height-adjustable posts, which allowed for precisely adjusting the distance (in the z-direction) between the donor and receiver while maintaining the parallelism of the two substrates. In the experiments, the distance between the donor and receiver substrates was typically set to a few 100 µm. This customized LIFT stage was mounted onto the movable table of the microstructuring system, which could adjust the position of the donor (in the x- and y-directions) relative to the scan field of the laser. With this combination of movable stages, unused areas of the donor could be located for each new laser pulse, even when a close or identical receiver location was addressed with subsequent laser pulses.

### 2.2. Preparation of Donor and Receiver Substrates

As donor substrate material, 4” borosilicate glass wafers (700 μm Borofloat^®^ substrates from Schott AG, Mainz, Germany), which are transparent to radiation at 1030 nm and 515 nm, were used. The Au transfer material was deposited via magnetron sputtering onto the glass wafer (Laborsystem LS 440 S, Ardenne Anlagentechnik GmbH, Dresden, Germany) after the wafer was cleaned in a spray processor (Acid Cleaner Convac MDL 1110SN175, Fairchild Semiconductor, San José, CA, USA) and dried under ambient air on a hotplate at 120 °C. It was found in the first LIFT experiments that the coating quality heavily depends on sufficient dehumidification of wafer substrates. Therefore, the substrates had to remain for at least 2 h on the hotplate right before sputter coating. The deposition was performed at a power of only 100 W to reduce the sputter rate and allow for more precise control of the layer thickness. It was observed that while the sputter target changed over time due to progressing abrasion, the deposition rate also slightly varied. Therefore, the layer thickness was measured using two different methods. Tear-off edges were created using adhesive tape, which was used to remove the deposited layer at the edge of the wafer. The height of the resulting step (i.e., layer thickness) was measured using either a laser scanning microscope (VK-X260K, Keyence, Osaka, Japan) or a stylus profilometer (Dektak 8 Stylus Profiler, Veeco Instruments Inc., Plainview, NY, USA). The average was taken from measurements at five positions along the tear-off edge, with a distance of 10 mm from each other. In addition, a non-destructive method for weighing the wafer before and after the sputtering process was used to determine the average layer thickness by assuming a specific density of 19.32 g/cm^3^ for Au. Due to the deposition characteristics of the magnetron sputter coater, the layers were a few percent thicker in the middle of the wafer than at the edges. By tracking the deposition rates with progressing erosion of the target, the deposition times could be adjusted accordingly. For the parameter evaluation, silicon wafer pieces coated with parylene C (Parylene P6, Diener Electronic, Ebhausen, Germany) with a layer thickness of 10 µm were used as receiver substrates. The porous electrodes were fabricated on glass receiver substrates (same wafer material as used for the donor) covered with a polymer tape that was cut using a CO_2_ laser as a shadow mask before a first 30 nm-thick layer of Cr and a second 900 nm-thick layer of Au without masking was deposited using a sputtering process (Laborsystem LS 440 S, Von Ardenne Anlagentechnik GmbH).

### 2.3. Parameter Evaluation for the LIFT Process

Five different donors were prepared with Au thicknesses ranging from 291 to 318 nm (standard derivation of ±10 nm) because the layer thickness was expected to have a critical influence on the transfer behavior sketched in Figure 1 The pulse energies and the z-height (the gap between donor and acceptor) were varied systematically in order to identify suitable operation windows for the LIFT process. To investigate the influence of the focus, a test pattern combining different focus positions with different pulse energies was used.

### 2.4. Printing Strategies

The positioning and sequence in which the voxels arrive at the donor during the printing are of crucial importance. The voxel material leaves the donor layer in a partially or completely molten state and solidifies in an almost spherical form when arriving at the receiver substrate. The materials from subsequently transferred voxels can adhere to each other since the gold is partially melted when it hits the receiver and earlier printed voxel volumes. In order to achieve a distribution of voxels leading to a porous arrangement, a special printing strategy needed to be developed. The repetition rate of voxels to be printed corresponds to the pulse frequency of the laser, which could be adjusted up to 600,000 pulses per second in this setup. In order to create a closed conductive surface, neighboring voxels should overlap so that the corresponding transferred voxel material comes in contact. This was achieved with a pitch of 3–4 µm for voxels with diameters of about 5 µm. On the donor, the transfer material was addressable in the form of a thin solid film. The diameter of the irradiated disk at the donor was therefore much larger than the diameter of the almost spherical voxel on the receiver and the distance from one voxel to the next must be larger on the donor side to avoid overlapping, which would lead to disruption of the voxels. In this work, the focused beam diameter was measured to be 25 µm and a minimum pitch of 30 µm in the donor was therefore kept. As a consequence of this and because LIFT-printed unit cells consist of stacked layers, the donor had to be moved independently from the receiver to address an area on the donor that was approximately 100 times larger than the printed conducted surface area on the receiver.

**Figure 3:**
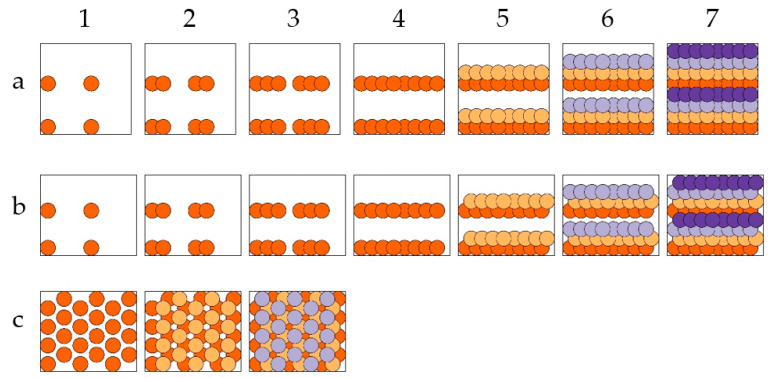
Illustration of three different printing strategies. Circular areas represent the voxels with the respective x/y-positions on the receiver substrate. Steps 1 to 7 illustrate the sequence in which overlapping voxels were printed, leading to three different arrangements of unit cells: (**a**) with four layers of two-row prints, each consisting of 16 voxels; (**b**) with four staggered layers of two-row prints, each consisting of 16 voxels; (**c**) with three staggered layers of hexagonally ordered prints consisting of 20 or 24 voxels. Voxels of the same layer are shown in an identical color.

Different printing strategies with unit cells filled with layers of 64 voxel positions are illustrated in Figure 3. All rectangular unit cells covered the same acceptor area. Row-by-row printing resulted in the stacking of voxel elements and staircase-like structures within one layer (Figure 3a,b). To reduce the staircase formation, a third printing strategy, which retained the hexagonal pattern in the unit cell but adjusted the order of the printed voxels, was also used (Figure 3c) in the experiments. Here, the unit cell was divided into three sequentially printed layers, in each of which no voxel touched any neighbor. This should result in a more homogeneous porosity. The LIFT printing using these three printing strategies was followed by an inspection with a scanning electron microscope (Phenom XL, Thermo Fisher Scientific Inc., Waltham, MA, USA). Using a focused electron and ion beam combination (Helios G4 CX DualBeam system, FEI Company, Hillsboro, OR, USA), cross-sections were also prepared and investigated using samples that were LIFT-printed with the finally chosen parameters.

### 2.5. Circular Electrode Printing for Electrochemical Test

**Figure 4:**
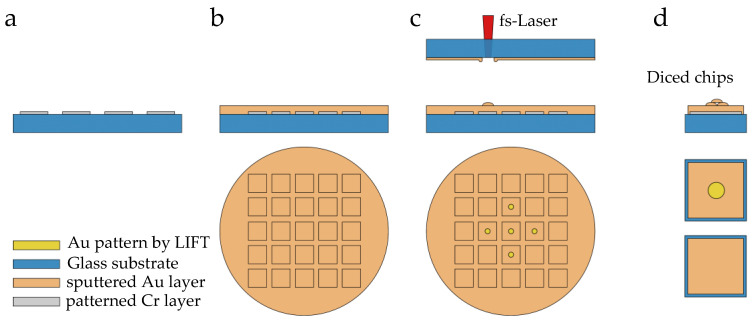
Schematic illustration of LIFT-printed ordered porous gold electrode fabrication: (**a**) sputtered Cr adhesion layer, (**b**) continuous Au layer and (**c**) LIFT deposition of the Au pattern. Planar Au layer without LIFT deposition was taken as the reference material. (**d**) Square chips with and without porous structures were obtained after dicing.

The patterned Cr layer for promoting adhesion (Figure 4a) was covered with a continuous Au layer (Figure 4b). Even though each layer thickness was precisely defined, it was difficult to avoid uncertainness in the substrate tilt and bow, and thus, a procedure was required to find the z-distance at which the focus was precisely located at the glass–metal interface of the donor. After evaluating the variation in the z-distance and laser power combinations, the conditions for the desired gold structures were found where droplet-like shapes were transferred as voxels of the printed porous Au electrodes. With the previously described hexagonal printing strategy, an accumulation of the gold particles as 3D ordered porous squares as unit cells (3 µm × 3 µm) was printed. By repeating unit cells, circular 3D-printed porous electrodes with a diameter of 3 mm were located in the center of the chip areas (Figure 4c). The wafer was finally diced (DAD 320 Dicing Saw from DISCO Corporation) to obtain separate 1 cm × 1 cm square chips with and without (for use as gold reference material) porous structures (Figure 4d). In view of the sensitivity of electrochemical measurements to impurities, samples were cleaned with piranha acid (3:1 of concentrated sulphuric acid and 30% hydrogen peroxide solution) and the diced chips were stored in a vacuum and dry environment to avoid contamination on the Au surface.

### 2.6. Electrochemical Setup

Electrochemical experiments were performed in a wall-jet setup including a three-electrode configuration under the static mode, i.e., without an electrolyte flow. The general design was based on previous work by Temmel et al. [16].

Mercury–mercury oxide (MMO) was used as the reference electrode and a Pt mesh as the counter electrode. Freshly prepared 0.1 M KOH (99.98% metal basis, AlfaAesar) solution was used as the electrolyte solution and saturated with Argon (5.0 quality, Westfalen AG, Münster, Germany) for at least 30 min. Applied potentials were iR-corrected and converted to the reversible hydrogen electrode (RHE) potential scale, respectively.

Cyclic voltammograms were recorded at a scan rate ν=50 mV/s between 0.05 and 1.50 V vs. RHE. The geometric surface area $A_{\mathrm{geo}}$ of ${0.163\;\mathrm{cm}}^{2}$ was based on the area defined by the O-ring, which was sealed on top of the electrode inside the wall-jet flow cell (see [16] for a schematic overview). The electrochemically active surface area (SA in cm^2^) was determined using the charge associated with the reduction of a gold oxide monolayer $Q_{\exp}$ via the following equation:

(1) $$\mathrm{SA}=\frac{Q_{\exp}}{\nu \;\cdot {\;Q}_{\mathrm{pseudo}}}$$

where $Q_{\mathrm{pseudo}}$ is the pseudocapacity of the oxide monolayer on a polycrystalline gold surface ($Q_{\mathrm{pseudo}}=390\;µC/\mathrm{cm}²$) [17]. The roughness factor rf of the electrode is then given by

(2) $$\mathrm{rf}=\frac{\mathrm{SA}}{A_{\mathrm{geo}}}$$

## 3. Results and Discussion

### 3.1. Optimization of the LIFT Printing Process of Single Voxels

**Figure 5:**
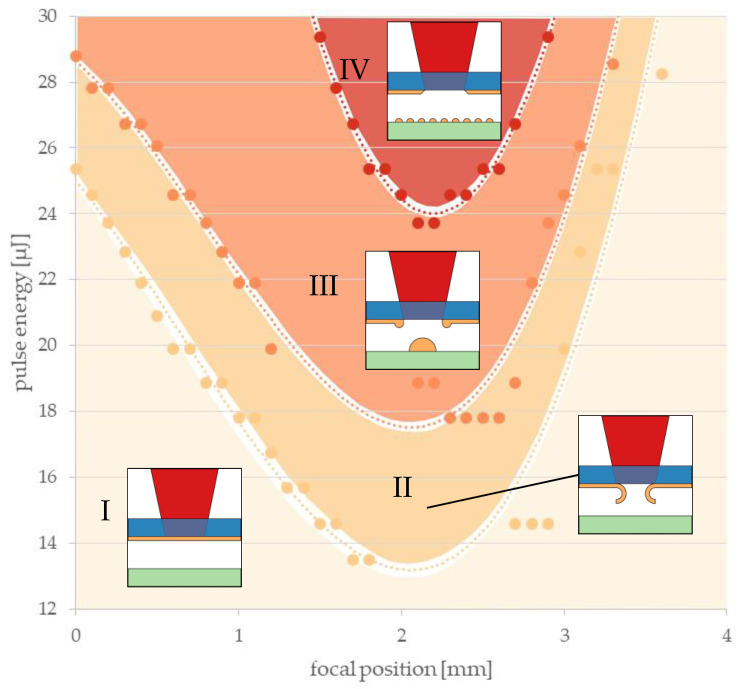
Exemplary result of the microscopic evaluation of test patterns, which were printed using LIFT with a gold donor layer of 291 nm and a z-height of 245 µm. Data points represent observed transitions that depended on the pulse energies and focal positions. Dotted lines represent polynomial approximations of the data points marking transitions. Four different regimes were identified and are separated by these lines: (I) donor is not affected; (II) donor is affected, but no transfer; (III) LIFT leads to voxel formation; (IV) evaporation and deposition as random small particles (fragments of voxels).

Figure 5 shows an example evaluation of LIFT transfer tests obtained with a donor layer thickness of 291 ± 10 nm and a z-height of 245 nm. A wavelength of 1030 nm was chosen with a 1.5 mm diameter pinhole for the beam shaping. Different regimes that depended on the focal position and pulse energy were identified, as illustrated Figure 5.

Dotted curves represent the thresholds between these characteristic regimes, which were determined via a microscopic evaluation of the donor and receiver substrates after the LIFT experiments. In regime I, the energy density was too low and no change was observed in the donor or receiver. Regime II signifies that the metal appeared locally delaminated from the donor substrate, but no transferred metal could be found on the receiver with the microscope. Only in regime III was the energy density suitable to reliably transmit voxels to the receiver with each laser pulse. Due to excessive laser energy, vaporization of the voxel material predominated in region IV and, therefore, the transmission of individual voxels was no longer possible. Only traces of evaporated material could be found on the receiver. For all threshold curves, a minimum was observed at a focal position of 2 mm where the laser focus was placed right within the donor metal layer so that the energy density in the donor metal was maximized. Similar evaluation diagrams were obtained for all investigated combinations of gold layer thickness, z-height, wavelength and beam setup. Generally, this setup enabled the transmission of voxels in a recognizable range of pulse energies and focal positions.

**Figure 6:**
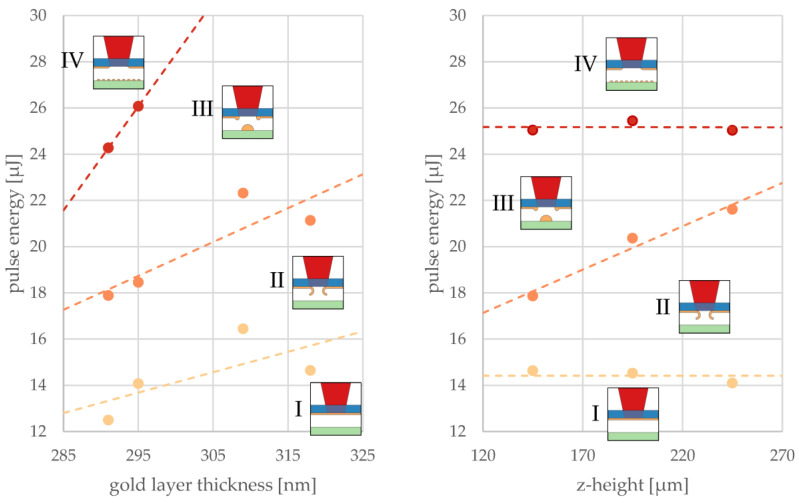
Influence of the layer thickness (**left**) and gap distance in the z-direction (**right**) on the minimum pulse energy marking the transition between the LIFT printing regimes I–IV introduced in Figure 5. Each data point represents the pulse energy minimum averaged over the z-heights (left) for one layer thickness or averaged over different layer thicknesses for one z-height (right). Straight lines serve as a guide for the eye to distinguish between the different regimes.

In the following, only the minima of polynomial fits to data points that separate the four different reaction zones (as displayed in Figure 5) were considered. In other words, precise focusing of the laser beam on these minimum pulse energies of the respective thresholds was assumed. Two graphs in Figure 6 illustrate these minima that mark transitions between the described regimes as obtained with varied gold layer thicknesses and z-heights. The data points represent pulse energy thresholds averaged over the z-heights (Figure 6, left panel) or averaged over different layer thicknesses (Figure 6, right panel).

The thresholds for affected donor and voxel printing increased with the thickening of donor layers because more energy deposition within the donor was required but light absorption was not increasing at the same rate. Therefore, a linear dependency of thresholds on the gold layer thickness could be assumed. The threshold for evaporation could not be recorded for larger layer thicknesses of 309 and 318 nm because our experiments did not allow for a pulse energy greater than 29 µJ.

The gap to the receiver substrate had no influence on the thresholds in the donor transitions between regimes I and II (yellow dashed line), as well as between III and IV (red dashed line), as can be seen from the constant pulse energies at different z-heights (Figure 6, right panel). However, the threshold for voxel printing was found to rise with z-height. It can be speculated that the liquid metal formed a jet that had a certain length depending on available energy and may have snapped back to the donor when it did not reach the receiver substrate. At the extrapolated crossing of thresholds II to III and III to IV (at about 325 µm), regime III disappeared and the LIFT voxel transfer was no longer possible. At the extrapolated crossing of thresholds from I to II and II to III (at about 0 µm), regime II disappeared and the LIFT voxel transfer was limited by jet formation.

Due to inhomogeneities in the sputter deposition, very few unexpected transitions between the zones seemed to randomly occur that could complicate the LIFT printing process. In summary, the best strategy can be described as follows: energy thresholds and focal distances for each donor wafer and each setting should be determined before the pulse energies for the printing of electrodes are selected in the transitions from thresholds II to III and III to IV.

### 3.2. Printing Strategies

**Figure 7:**
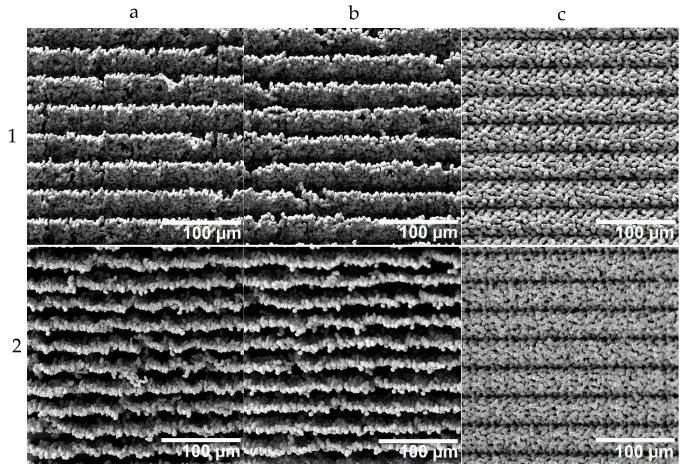
Scanning electron microscopy (SEM) images of structures obtained with different printing strategies (**a**–**c**) corresponding to the printing strategies a, b and c illustrated in Figure 3. The SEM micrographs in row 1 show top views, while the SEM micrographs in row 2 show views with a tilt of 45°. Columns (**a**–**c**) correspond to the three different printing strategies sketched in Figure 3.

With the knowledge of how to print precisely positioned individual voxels and with repeatable quality, the construction of surfaces and structures was investigated. For this purpose, a square surface with an edge length of 1 mm was printed using the different strategies discussed above. The LIFT printing was evaluated based on scanning electron micrographs, as shown in Figure 7.

The stair formation resulting from the two row-by-row strategies could be confirmed, and in the tilted view, even the bottom (surface of the acceptor substrate) became visible. The rows had no physical contact with each other; they were only connected through the ground, and thus, these two printing strategies were found to be less suitable. Especially considering that chips with applied LIFT structure need to be diced with a wafer saw, these structures appear unlikely to survive this intervention. Only the three-layer strategy resulted in well-connected and mechanically robust porous structures.

### 3.3. Printing of Electrodes

**Figure 8:**
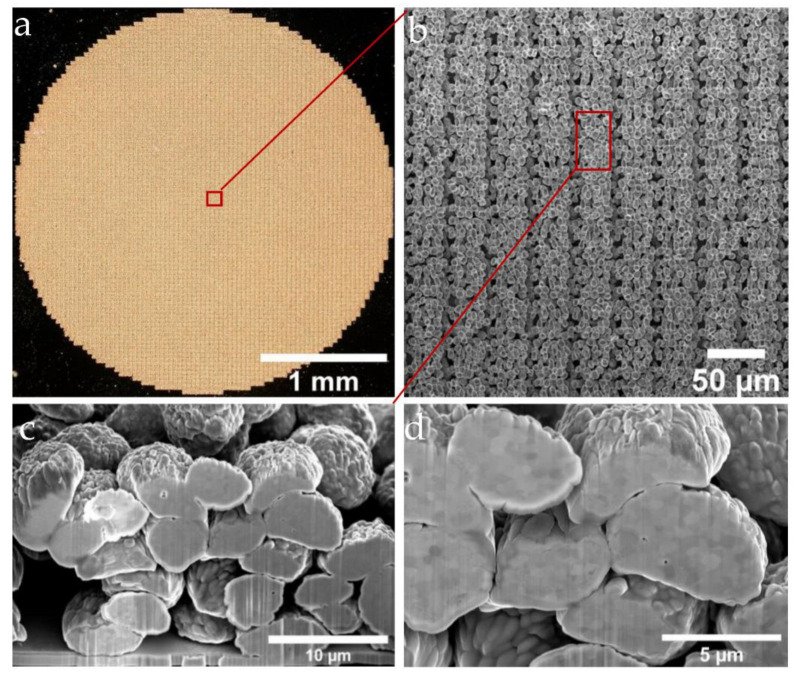
Microscope and SEM micrographs of the porous Au pattern with 30 µm in thickness with the hexagonal three-layer printing strategy (the experimental parameters: wavelength of 1030 nm, with z-height of 220 µm, a donor thickness of 300 nm and a 900 nm sputtered Au film as the contact layer on top of a receiver glass substrate). (**a**) Illustration of the surface morphology of the circular electrode. (**b**) A further enlarged image showing the morphology of each printed segment. The FIB milling area is shown in red. (**c**) Zoomed-in cross-sectional image showing the surface and cross-sectional details of the voxels. (**d**) A 52° tilted cross-sectional image of a 3D electrode after polishing with the FIB.

Using the three-layer strategy sketched in Figure 3c, porous electrodes were LIFT-printed, as shown in Figure 8.

A circular electrode was LIFT-printed with an adaptation to the electrochemical setup (optical micrograph in Figure 8a). By means of SEM, the morphologies of the LIFT-printed electrodes were evaluated. It can be seen that although the gold particles had an approximate diameter of 5 µm and a voxel-to-voxel pitch of around 3 µm, which showed some small degree of local randomness, the structure of the electrode showed a clear repeating pattern (Figure 8b). To investigate the porosity of the electrodes and the stacking characteristics, as well as the connections between the voxel elements, cross-sectional views were obtained via FIB preparation (Figure 8b, red region). With a sample tilt of 52°, the cross-section of the printed unit cell could be investigated by using SEM (Figure 8c,d). Based on the SEM data, the gold particle areas and the empty areas (Figure 8d) were evaluated using the image processing software Fiji (IMAGEJ, National Institutes of Health, Bethesda, MD) to determine a first estimate of the porosity of about 30%, which can be considered as representative for the sample with a deterministic pore structure.

### 3.4. Electrochemically Active Surface Area and Roughness Factor

**Figure 9:**
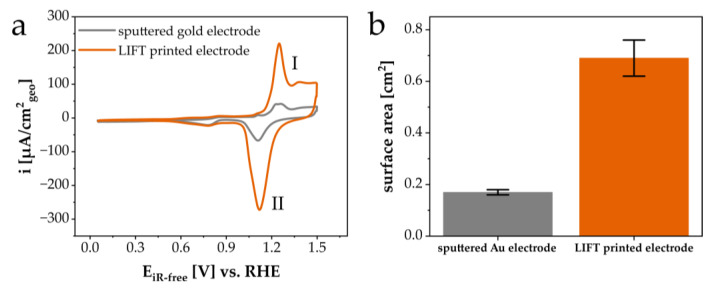
(**a**) Cyclic voltammetry (CV) profiles and (**b**) the corresponding electrochemically active surface areas (SA) of the LIFT-printed gold electrode (orange) and sputtered gold electrode (grey). The CV profiles were recorded at a scan rate of 50 mV/s in Ar-saturated 0.1 M KOH.

The applicability of the LIFT structures as porous electrode materials was evaluated by recording the cyclic voltammetry (CV) profiles in Ar-saturated 0.1 M KOH. Figure 9a displays the CV profile of a LIFT-printed electrode, showing the characteristic features of a clean Au surface, such as the formation (I) (1.0–1.5 V vs. RHE) and the reduction of a monolayer of Au-O (II) (1.3–1.0 V vs. RHE). The sputtered Au film was taken as the reference material. It was obvious that the geometrically normalized current densities of the oxidation and reduction process of Au-O for the LIFT-printed electrode were clearly higher compared with the sputtered gold electrode, indicating an increase in the electrochemically active surface area (SA). Figure 9b shows the resulting SA for both Au electrodes established using equation 1. The SA values of the LIFT-printed electrode were around 0.69 ± 0.07 cm^2^, and thus, around four times higher than for the sputtered Au (0.17 ± 0.01 cm^2^). According to equation 2, the values of the roughness factor (rf) were determined to be 4.2 and 1.0 for the LIFT-printed and sputtered Au electrodes, respectively. Other studies about macroporous or flat gold electrodes had similar rf values [18,19,20,21].

A follow-up work will focus on the optimization of the pore structure and the application of such LIFT-printed electrodes for electrochemical reactions, such as methanol oxidation reaction (MOR) in alkaline media. For the first time, it was demonstrated that the 3D LIFT structure led to an increase in the rf compared with a sputtered film.

## 4. Conclusions

We successfully developed a LIFT process using a femtosecond laser and a motorized custom-built stage to print 3D metal voxels on solid surfaces to form porous metal films. A parameter study methodology was developed that allowed for quickly and reliably finding the appropriate parameters for voxel printing. By programming the LIFT stage, different printing strategies for porous films could be realized. As proof of concept, we successfully printed 3D porous circular gold electrodes with a hexagonal three-layer print strategy. The increase in the surface area of the LIFT-printed Au materials based on the porosity and hierarchical arrangement was confirmed using electrochemical experiments in Ar-saturated 0.1 M KOH. The LIFT-printed Au electrode exhibited a four-fold higher electrochemically active surface area (SA) compared with a flat Au surface. This demonstrated that our approach could be considered very promising and stimulates future research to explore the full potential of this hierarchical ordered and porous electrode fabrication technique. For example, changing the focus level of the laser could provide the ability to print other voxel shapes and the number of layers could also be varied. Further, optical beam shaping could provide the ability to print much smaller voxels with an even higher SA. The application of such electrodes for electrochemical reactions, such as methanol oxidation reaction (MOR) in alkaline media, will be further studied.

## Data Availability

The test data generated during the experiments can be requested from the authors.

## Abbreviations

The following abbreviations are used in this manuscript:

LIFT	Laser-induced forward transfer
SA	Electrochemically active surface area
CV	Cyclic voltammetry
DMLM	Direct metal laser melting
MMO	Mercury–mercury oxide
RHE	Reversible hydrogen electrode
rf	Roughness factor
SEM	Scanning electrode microscope
MOR	Methanol oxidation reaction

## References

[1] Karim W., Tschupp S.A., Oezaslan M., Schmidt T.J., Gobrecht J., van Bokhoven J.A., Ekinci Y. (2015). High-resolution and large-area nanoparticle arrays using EUV interference lithography. Nanoscale.

[2] Hedayat N., Du Y., Ilkhani H. (2017). Review on fabrication techniques for porous electrodes of solid oxide fuel cells by sacrificial template methods. Renew. Sustain. Energy Rev..

[3] Vu A., Qian Y., Stein A. (2012). Porous Electrode Materials for Lithium-Ion Batteries—How to Prepare Them and What Makes Them Special. Adv. Energy Mater..

[4] Arenas L.F., Ponce de León C., Walsh F.C. (2017). 3D-printed porous electrodes for advanced electrochemical flow reactors: A Ni/stainless steel electrode and its mass transport characteristics. Electrochem. Commun..

[5] Arenas L.F., Ponce de León C., Walsh F.C. (2019). Three-dimensional porous metal electrodes: Fabrication, characterisation and use. Curr. Opin. Electrochem..

[6] Patel J., Radhakrishnan L., Zhao B., Uppalapati B., Daniels R.C., Ward K.R., Collinson M.M. (2013). Electrochemical properties of nanostructured porous gold electrodes in biofouling solutions. Anal. Chem..

[7] Zhao B., Collinson M.M. (2010). Well-defined hierarchical templates for multimodal porous material fabrication. Chem. Mater..

[8] Szamocki R., Velichko A., Holzapfel C., Mücklich F., Ravaine S., Garrigue P., Sojic N., Hempelmann R., Kuhn A. (2007). Macroporous ultramicroelectrodes for improved electroanalytical measurements. Anal. Chem..

[9] Szamocki R., Velichko A., Mücklich F., Reculusa S., Ravaine S., Neugebauer S., Schuhmann W., Hempelmann R., Kuhn A. (2007). Improved enzyme immobilization for enhanced bioelectrocatalytic activity of porous electrodes. Electrochem. Commun..

[10] Qi Z., Hawks S.A., Horwood C., Biener J., Biener M.M. (2022). Mitigating mass transport limitations: Hierarchical nanoporous gold flow-through electrodes for electrochemical CO_2_ reduction. Mater. Adv..

[11] Piqué A., Serra P. (2018). Laser Printing of Functional Materials: 3D Microfabrication, Electronics and Biomedicine.

[12] Zenou M., Kotler Z. (2016). Printing of metallic 3D micro-objects by laser induced forward transfer. Opt. Express.

[13] Luo J., Pohl R., Qi L., Römer G.-W., Sun C., Lohse D., Visser C.W. (2017). Printing Functional 3D Microdevices by Laser-Induced Forward Transfer. Small.

[14] Kuznetsov A.I., Kiyan R., Chichkov B.N. (2010). Laser fabrication of 2D and 3D metal nanoparticle structures and arrays. Optics Express.

[15] Hecht L., Rager K., Davidonis M., Weber P., Gauglitz G., Dietzel A. (2019). Blister-actuated LIFT printing for multiparametric functionalization of paper-like biosensors. Micromachines.

[16] Temmel S.E., Tschupp S.A., Schmidt T.J. (2016). A highly flexible electrochemical flow cell designed for the use of model electrode materials on non-conventional substrates. Rev. Sci. Instrum..

[17] Trasatti S., Petrii O.A. (1991). Real surface area measurements in electrochemistry. Pure Appl. Chem..

[18] Zhao B., Collinson M.M. (2012). Hierarchical porous gold electrodes: Preparation, characterization, and electrochemical behavior. J. Electroanal. Chem..

[19] Jarzabek G., Borkowska Z. (1997). On the real surface area of smooth solid electrodes. Electrochim. Acta.

[20] Szamocki R., Reculusa S., Ravaine S., Bartlett P.N., Kuhn A., Hempelmann R. (2006). Tailored Mesostructuring and Biofunctionalization of Gold for Increased Electroactivity. Angew. Chem. Int. Ed.

[21] Reculusa S., Heim M., Gao F., Mano N., Ravaine S., Kuhn A. (2011). Design of Catalytically Active Cylindrical and Macroporous Gold Microelectrodes. Adv. Funct. Mater..

